# Association of cardiovascular disease and 10 other pre-existing comorbidities with COVID-19 mortality: A systematic review and meta-analysis

**DOI:** 10.1371/journal.pone.0238215

**Published:** 2020-08-26

**Authors:** Paddy Ssentongo, Anna E. Ssentongo, Emily S. Heilbrunn, Djibril M. Ba, Vernon M. Chinchilli

**Affiliations:** 1 Department of Public Health Sciences, Penn State College of Medicine, Hershey, Pennsylvania, United States of America; 2 Center for Neural Engineering, Department of Engineering, Science and Mechanics, The Pennsylvania State University, University Park, Pennsylvania, United States of America; 3 Department of Surgery, Penn State College of Medicine and Milton S. Hershey Medical Center, Hershey, Pennsylvania, United States of America; University of Oxford, UNITED KINGDOM

## Abstract

**Background:**

Estimating the risk of pre-existing comorbidities on coronavirus disease 2019 (COVID-19) mortality may promote the importance of targeting populations at risk to improve survival. This systematic review and meta-analysis aimed to estimate the association of pre-existing comorbidities with COVID-19 mortality.

**Methods:**

We searched MEDLINE, SCOPUS, OVID, and Cochrane Library databases, and medrxiv.org from December 1^st^, 2019, to July 9^th^, 2020. The outcome of interest was the risk of COVID-19 mortality in patients with and without pre-existing comorbidities. We analyzed 11 comorbidities: cardiovascular diseases, hypertension, diabetes, congestive heart failure, cerebrovascular disease, chronic kidney disease, chronic liver disease, cancer, chronic obstructive pulmonary disease, asthma, and HIV/AIDS. Two reviewers independently extracted data and assessed the risk of bias. All analyses were performed using random-effects models and heterogeneity was quantified.

**Results:**

Eleven pre-existing comorbidities from 25 studies were included in the meta-analysis (n = 65, 484 patients with COVID-19; mean age; 61 years; 57% male). Overall, the between-study heterogeneity was medium, and studies had low publication bias and high quality. Cardiovascular disease (risk ratio (RR) 2.25, 95% CI = 1.60–3.17, number of studies (n) = 14), hypertension (1.82 [1.43 to 2.32], n = 13), diabetes (1.48 [1.02 to 2.15], n = 16), congestive heart failure (2.03 [1.28 to 3.21], n = 3), chronic kidney disease (3.25 [1.13 to 9.28)], n = 9) and cancer (1.47 [1.01 to 2.14), n = 10) were associated with a significantly greater risk of mortality from COVID-19.

**Conclusions:**

Patients with COVID-19 with cardiovascular disease, hypertension, diabetes, congestive heart failure, chronic kidney disease and cancer have a greater risk of mortality compared to patients with COVID-19 without these comorbidities. Tailored infection prevention and treatment strategies targeting this high-risk population might improve survival.

## Introduction

The number of total cases of the coronavirus-2019 disease (COVID-19) continues to rise quickly, threatening thousands to millions of individuals with pre-existing chronic conditions who are disproportionately affected [1]. To date, as of July 9^th^, 2020, the Johns Hopkins University coronavirus resource center reported that worldwide more than 180 countries have been affected with COVID-19 with more than twelve million confirmed cases and more than 500,000 deaths. [2] As research related to potential risk factors for COVID-19 mortality continues, it is becoming clear that individuals with underlying comorbidities, such as cardiovascular diseases, hypertension, diabetes, congestive heart failure, cerebrovascular disease, chronic kidney disease, chronic liver disease, cancer, chronic obstructive pulmonary disease, asthma, and HIV/AIDS, may have a greater risk of death from COVID-19. [3, 4] As the number of published studies increases, there is a widening gap due to inconsistent findings related to the influence of pre-existing comorbidities on COVID-19 mortality. Some studies report an association between pre-existing conditions and COVID-19 mortality, whereas others report no association. The differences may stem from the limited number of studies analyzed, heterogeneous methodologies and unaddressed sources of bias. Nevertheless, it is clear that regions experiencing the highest mortality rates, such as the United States, Europe, and China, also have the greatest burden of these pre-existing chronic conditions. [5]

The novel virus, severe acute respiratory syndrome coronavirus 2 (SARS-CoV-2), the causative agent of COVID-19, interacts with angiotensin-converting enzyme 2 (ACE2), a cellular binding site expressed in the heart, kidney and pulmonary alveolar type II cells. [6] It has been postulated, though not confirmed, that pre-existing use of angiotensin II type 1 receptor blockers (ARBs) may upregulate membrane-bound ACE2 hence increasing susceptibility to virus entry in humans. [7] Therefore, it is plausible that individuals with pre-existing chronic conditions such as hypertension and chronic heart failure taking ARBs may be more susceptible to the severity of SARS-CoV-2, including mortality.

To date, studies that have systematically explored the association of a range of pre-existing chronic conditions and COVID-19 mortality have limitations in the number of countries included, the number of studies included, and the number of conditions explored. [8, 9]. Furthermore, these studies have significant unaddressed sources of bias that limit conclusions drawn from them. We took a comprehensive approach and estimated the association of major pre-existing chronic conditions, including cardiovascular diseases, hypertension, diabetes, congestive heart failure, cerebrovascular disease, chronic kidney disease, chronic liver disease, cancer, chronic obstructive pulmonary disease, asthma, and HIV/AIDS, and the risk of mortality from COVID-19. Although the majority of studies occurred in China, we identified additional studies involving patients from Europe, North America, and Africa.

## Methods

### Information source and search strategy

The present study has been registered with PROSPERO (registration ID: CRD42020187972). This study is being reported in accordance with the reporting guidance provided in the Preferred Reporting Items for Systematic Reviews and Meta-Analyses (PRISMA) statement and Meta-analysis of Observational Studies in Epidemiology (MOOSE). [10, 11] We searched PubMed (MEDLINE), OVID (MEDLINE, HEALTHSTAR), SCOPUS, Joana Briggs International EBP, and Cochrane Library databases (from December 1^st^, 2019, and July 7^th^, 2020). We searched the grey or difficult to locate literature, including Google Scholar and Medrxiv. We performed hand-searching of the reference lists of included studies, relevant reviews, or other relevant documents. Studies reporting the risk of mortality in patients with COVID-19 were included. No limitations were applied on study design, country of publication, or language. Non-English-language articles were translated using language translation services at Penn State University Library and further included in the screening. Predefined search terms determined by the Medical Subject Headings (MeSH) and keywords included multiple combinations of the following: (“COVID-19” OR “coronavirus” OR “SARS-CoV-2” OR "2019-nCoV" OR "SARS nCoV2",) AND (“mortality”) AND (“cardiovascular disease” OR “chronic obstructive pulmonary disease” OR “asthma” OR “coronary heart disease” OR “coronary artery disease” OR “hypertension” OR “diabetes” OR “congestive heart failure” OR “malignancy” OR “cancer” OR “chronic kidney disease” OR “chronic liver disease” OR “cerebrovascular disorders" OR "stroke “comorbidities” OR “asthma”, OR” “HIV/AIDS” OR” “human immunodeficiency virus infection AND acquired immune deficiency syndrome”). Two reviewers (ESH and AES) independently screened titles and abstracts of the studies for inclusion eligibility. The comprehensive list of studies found as a result of our initial search was transferred into Endnote, which further removed duplicate studies.

### Eligibility criteria

Studies were selected according to the following criteria: participants, exposure, comparison, condition or outcome(s) of interest, study design and context.

***1*. *Participants (population)*:** We included studies involving patients hospitalized for COVID-19, regardless of age.
***2*. *Exposure*:** The exposure included any of the 11 comorbidities including cardiovascular diseases, hypertension, diabetes, congestive heart failure, cerebrovascular disease, chronic kidney disease, chronic liver disease, cancer, chronic obstructive pulmonary disease, asthma, and HIV/AIDS.
***3*. *Comparison*:** Hospitalized patients with COVID-19 without the above-mentioned pre-existing comorbidities
***4*. *Condition or outcome(s) of interest*:** The primary outcome was the mortality in hospitalized patients with COVID-19 with comorbidities in comparison to hospitalized patients with COVID-19 without comorbidities.
***5*. *Study design and context*:** Eligible studies were randomized controlled trials, cohort (prospective or retrospective), case series (with at least 10 patients), and case-control studies.
  Criteria of inclusion included the following: COVID-19 diagnosis was based on the World Health Organization guidance; [12] studies examined the association of any of the pre-existing comorbidities and COVID-19; the risk point estimates reported as odds ratios (ORs), risk ratios (RRs), or hazard ratios (HRs) or the data was presented such that the OR, RR, and HR could be calculated; the 95% CI was reported, or the data were presented such that the 95% CI could be calculated. We excluded case reports or case series with less than 10 patients, studies not conducted on humans, review papers, meta-analyses, literature reviews, and commentaries. Excluded studies were documented with reasons for their exclusion.

### Data extraction

Two reviewers (ESH and AES) initially screened titles and abstracts of all identified articles for eligibility. After initially screening articles for inclusion based on titles and abstracts, full-text articles were screened. Disagreements were resolved by discussion to meet a consensus. If necessary, a third reviewer (PS) was consulted in order to reach a consensus. We extracted the following information: year of publication, date of the study, the sample size of patients with COVID-19, number of participants with each comorbidity who died and did not die, the point estimates and 95% confidence interval of mortality of patients with COVID-19 with each underlying condition (S1 Table), the mean or median age with their corresponding standard deviation or interquartile range, respectively, the proportion that was male, and the covariates adjusted for each study. We gave priority to adjusted estimates if available.

### Study quality assessment

Two reviewers (EH and AES) independently assessed the quality of the included studies. The Newcastle-Ottawa Scale (NOS) was utilized for the quality assessment of the included studies. [13] NOS scale rates observational studies based on 3 parameters: selection, comparability between the exposed and unexposed groups, and exposure/outcome assessment. It assigns a maximum of 4 stars for selection, 2 stars for comparability, and 3 stars for exposure/outcome assessment. Studies with less than 5 stars were considered low quality, 5–7 stars of moderate quality, and more than 7 stars of high quality. In reporting results, we gave preference for results from clinical trials over observational studies.

### Data synthesis and statistical analysis

We adopted a narrative approach to describing the number of studies, study settings, proportion of sex, mean or median age and covariates adjusted for in each study. Our primary outcome was the risk of mortality in patients with COVID-19 associated with pre-existing chronic diseases. We combined RRs and HRs with ORs in the present meta-analysis and reported the pooled effect size as RRs as common risk estimates for all studies.

For studies without measures of associations, a generalized linear mixed model was used to calculate the OR using the number of events and the sample size of each study group. [14]. Effect sizes were log-transformed to normalize the distributions. Next, standard errors (SEs) were calculated via the following equations [15]: Lower = log (lower 95% CI) and upper = log (upper 95% CI), and SE = (upper—lower)/3.92. To assess the associations between pre-existing conditions and the risk of mortality, we pooled the RR estimates for the presence versus absence of pre-existing conditions from each study, weighted by the inverse of their variances (inter-study plus intra-study variances). The *metagen* function from the R package meta was used to calculate the pooled effect estimates using random-effects models. [16] The DerSimonian and Laird (DL) random-effects method was used to estimate the pooled inter-study variance (heterogeneity). [17] We graphically displayed individual and pooled estimates with forest plots. Inter-study heterogeneity was assessed using *I*^2^ statistics, expressed as % (low (25%), moderate (50%), and high (75%) and Cochrane’s *Q* statistic (significance level < 0.05) [18, 19]. For outcomes with high between-study heterogeneity, influence sensitivity analysis was undertaken to explore the effect of each individual study on the overall pooled estimate. [20] If a specific comorbidity had 10 or more studies and significant between- study heterogeneity was observed, we conducted meta-regression analysis to explore sources of variation, using mean age and proportion of males as regressors. [16] Potential ascertainment bias (as might be caused by publication bias) was assessed with funnel plots, by plotting the study effect size against standard errors of the effect size, and Egger’s test. [21] Trim and fill analyses using Duval and Tweedie non-parameteric method were used to adjust for the publication bias. [22] All statistical analyses were performed with R software, version 3.4.3 (R, College Station, TX).

## Results

### Identified studies

**Fig 1:**
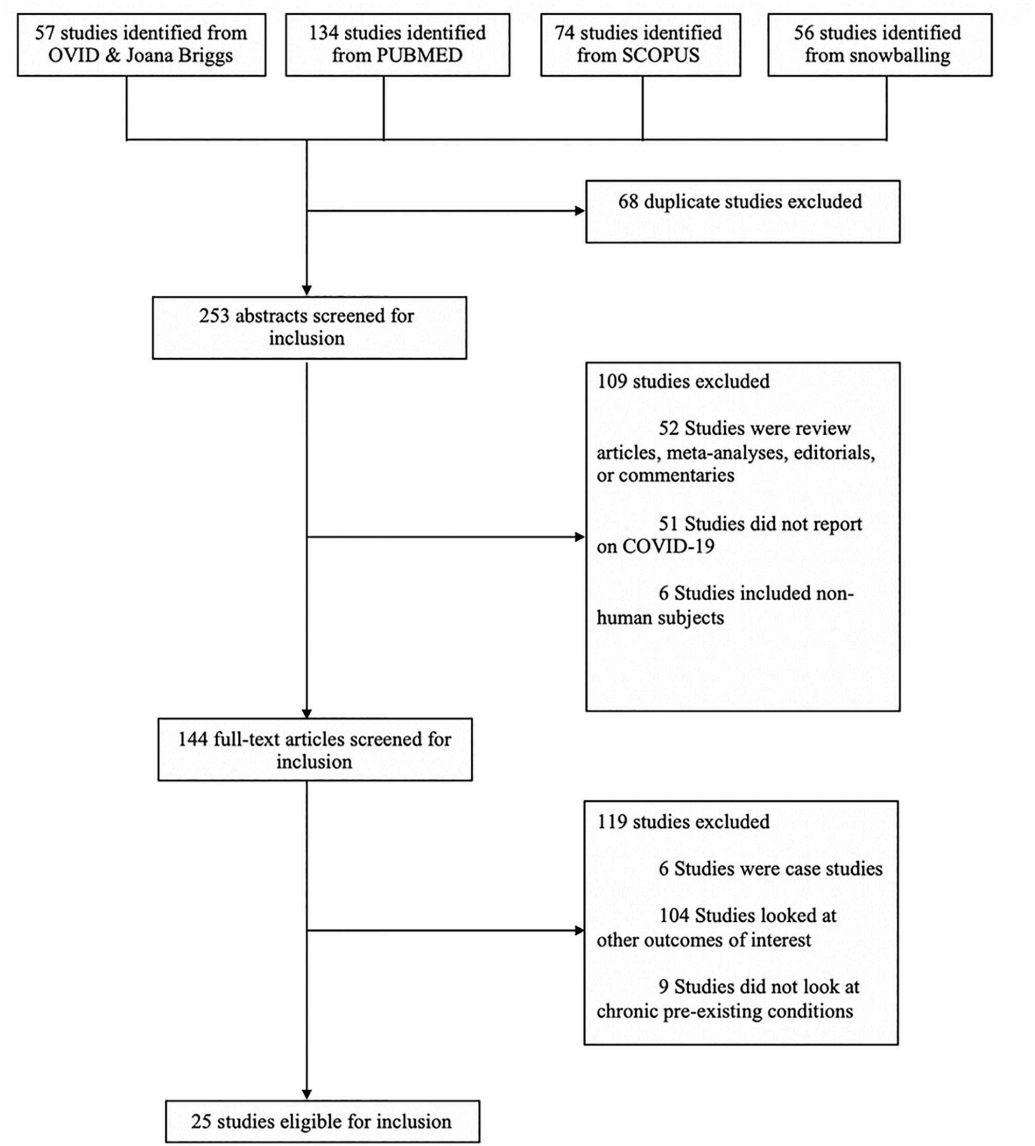
PRISMA flow diagram.

As shown in Fig 1, we identified a total of 321 studies. Of these, 68 studies were duplicates, leaving 253 studies to explore for inclusion. An additional 109 studies were excluded based on titles and abstracts and another 119 studies based on full text, which resulted in 25 studies for the quantitative analysis. The process yielded a total of 65, 484 patients with COVID-19 with 19 studies conducted in China, [3, 4, 23–39], 3 from the United States, [40–42] 1 in Italy, [43] and 1 in South Africa. [44] One study had 24 representative countries from North America, Europe, Asia, and Africa. [45] (Table 1). The mean age was 61 years and 57% were male. Median quality score was 7 (range = 5–9).

**Table 1:** Characteristics of studies included in the systematic review.

Author	Country	Sample size	Study type	Study period	Age, y	Male, N (%)	Covariates adjusted for	Quality Score
**Du et al** [23]	Wuhan Pulmonary Hospital, Wuhan, China	179	Prospective cohort	12/25/19-2/07/20	58 (Mean, SD: 13.7)	97 (54)	Age, cardiovascular or cerebrovascular disease, CD3+CD8+ T cells, Cardiac troponin I	8
**Zhou et al** [24]	Jinyintan Hospital and Wuhan Pulmonary Hospital, Wuhan, China	191	Retrospective cohort	12/29/19-1/31/20	56 (Median, Range: 46–67)	119 (62)	Age, coronary heart disease, SOFA score, lymphocyte, D-dimer	8
**Shi et al** [4]	Renmin Hospital of Wuhan University, Wuhan, China	416	Retrospective cohort	1/20/20-2/20/20	64 (Median, Range: 21–95)	205 (49)	Age, cardiovascular diseases, cerebrovascular diseases, diabetes, chronic obstructive pulmonary disease, renal failure, cancer, acute respiratory distress syndrome, cardiac injury, creatinine, terminal pro-B-type natriuretic peptide	9
**Li et al** [25]	Tongji Hospital, Wuhan, China	548	Retrospective cohort	1/26/20-2/05/20	60 (Median, Range: 48–69)	279 (51)	Age, sex, blood leukocyte count, LDH, complications (cardiac injury, hyperglycemia), corticosteroids (low dose, high dose), Lopinavir/Ritonavir,	9
**Guo et al** [26]	Seventh Hospital of Wuhan City, Wuhan, China	187	Retrospective case series	1/23/2022/23/20	59 (Mean, SD: 14.66)	91 (49)	-	6
**Grasselli et al** [43]	Fondazione IRCCS Ca’ Granda Ospedale Maggiore Policlinico, Milan, Italy	1,591	Retrospective cohort	2/20/20-3/18/20	63 (Median, IQR: 56–70)	1,304 (82)	-	6
**Chen et al** [3]	Tongji Hospital, Wuhan, China	274	Retrospective cohort	1/13/20-2/12/20	62 (Median, Range: 44–70)	171 (62)	-	5
**Fu et al** [27]	Union Hospital of Huazhong University of Science and Technology and Second Affiliated Hospital of Anhui Medical University, Wuhan, China	200	Case-cohort	1/01/20-1/30/20	-	99 (50)	Liver function indexes (alanine aminotransferase, total bilirubin), renal function (creatinine, urea nitrogen, uric acid), myocardial function (creatine kinase, myoglobin, lactate dehydrogenase, aspartate aminotransferase, aspartate aminotransferase/alanine aminotransferase ratio)	9
**Gu et al** [28]	Hubei Province, China	275	Nested case-control	12/18/19-3/08/20	66 (Mean, SD: 14.5)	173 (63)	Age, male, coronary heart disease, cerebral infarction, COPD, renal failure	7
**Yang et al** [29]	Wuhan Jin Yin-tan hospital, Wuhan, China	52	Retrospective cohort	12/24/20-1/26/20	60 (Mean, SD: 13.3)	35 (67)	-	7
**Deng et al** [30]	Hankou and Caidian branch of Tongji Hospital, Tongji Medical College, Huazhong University of Science & Technology, and Hankou branch of The Central Hospital of Wuhan, Wuhan, China	225	Retrospective cohort	1/01/20-2/21/20	-	-	-	6
**Yuan et al** [31]	Hubei Public Health Clinical Center, Wuhan, China	27	Retrospective cohort	1/01/20-1/25/20	60 (Median, Range: 47–69)	12 (45)	-	6
**Wang** [32] **et al**	Renmin Hospital of Wuhan University, Wuhan, China	339	Retrospective cohort	1/01/20-2/06/20	69 (Median, Range: 65–76)	166 (49)	Age, cardiovascular disease, cerebrovascular disease, COPD, acute cardiac injury, arrhythmia, AKI, ARDS, cardiac insufficiency, bacterial infection	9
**Li et al** [47]	Central Hospital of Wuhan, Wuhan, China	1,178	Retrospective case-series	1/15/20-3/15/20	66 (Median, Range: 59–73)	189 (52)	-	5
**Zhang et al** [33]	Tongji Sino-French New Town Hospital, Union Red Cross Hospital, and Union West Hospital, Wuhan, China	28	Retrospective cohort	1/13/20-2/26/20	65 (Median, Range: 56–70)	17 (61)	Sex, age	8
**Zhang et al** [48]	Wuhan No.1 Hospital, Wuhan, China	48	Retrospective cohort	12/25/20-2/15/20	71 (Mean, SD: 13.4)	33 (69)	Age, SpO2%, Serum Cr value, d-dimer value, hs-CTnI elevation	9
**Guan et al** [35]	575 hospitals, China	1,590	Retrospective cohort	12/11/20–1/31/20	49 (Mean, SD: 16.3)	904 (57)	Type of comorbidity (COPD, diabetes, hypertension, malignant tumor), number of comorbidities (1, 2 or more)	8
**Chen et al** [36]	Central Hospital of Wuhan, China	904	Retrospective cohort	1/1/20-3/17/20	56 (Median, Range: 39–67)	421 (47)	-	6
**Li et al** [38]	Wuhan Union Hospital, Wuhan, China	83	Retrospective cohort	2/1/20-2/20/20	43 (Median, Range: 32–62)	34 (41)	-	6
**Yang et al** [39]	Renmin Hospital of Wuhan University, Wuhan, China	52	Retrospective cohort	1/1/20-4/15/20	63 (Median, Range: 34–98)	28 (54)	-	7
**Cao et al** [37]	Wuhan University Zhongnan Hospital, Wuhan, China	102	Retrospective cohort	1/3/20-2/1/20	54 (Median, Range: 37–67)	53 (52)	-	7
**COVIDSurg Collaborative** [45]	International	1,128	Retrospective and prospective cohort	1/1/20-3/31/20	-	523 (46)	-	7
**Chhiba et al** [40]	10 hospitals affiliated with Northwestern Medicine, Illinois, USA	1,526	Retrospective cohort	3/1/20-4/15/20	Range 40–69	718 (47)	-	7
**Feng et al** [34]	China’s Infectious Disease Information System, China	44,672	Retrospective cohort	Reported through 2/11/20	-	22,981 (51.4)	-	7
**Davies et al** [44]	Western Cape Provincial Health Data Centre, Africa	22,308	Retrospective cohort	3/1/20-6/9/20	-	-	Age, sex, location	9
**Karmen-Tuohy et al** [41]	USA	63	Case-control	3/2/20-4/23/20	60 mean, SD (11)	57 (90)	-	7
**Marcello et al** [42]	USA	6248	Retrospective cohort	3/5/20-4/16/20	61 medians (50–73)	3851(62)		7

Abbreviations: COPD: Chronic obstructive pulmonary disease; ACE: Angiotensin-converting enzyme: ARB: Angiotensin II receptor blocker; hs-CTnI: high-sensitivity cardiac troponin I: AKI: Acute kidney injury; ARDS: Acute respiratory distress syndrome; SOAF: sequential organ failure assessment; LDH: Lactate dehydrogenase.

### The risk of mortality in patients with COVID-19 with cardiovascular disease

**Fig 2:**
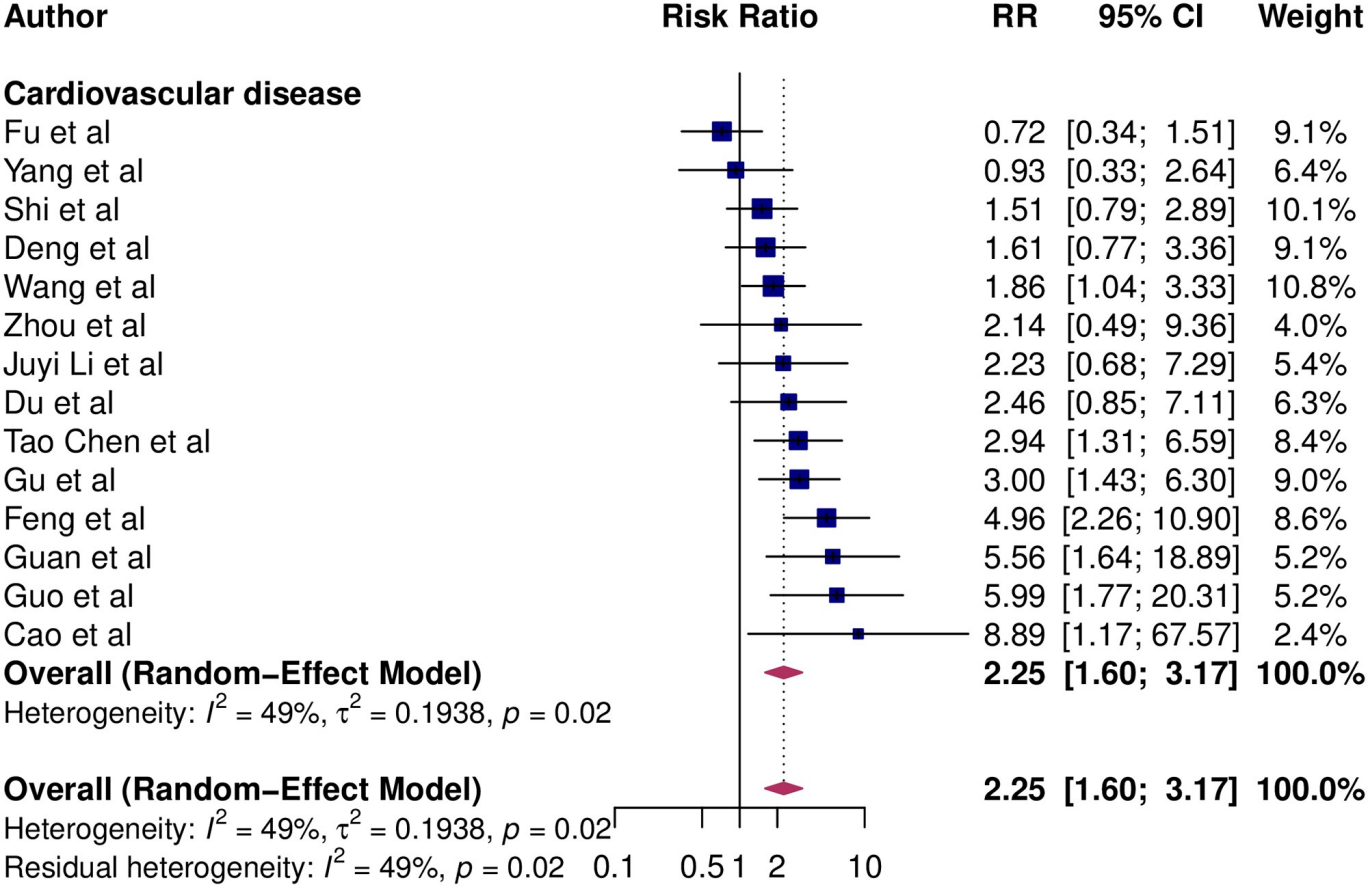
Association of cardiovascular disease and mortality risk from COVID-19. Blue squares and their corresponding lines are the point estimates and 95% confidence intervals per each study. Maroon diamond represents the pooled effect estimate.

Of the fourteen studies that reported the risk of mortality associated with pre-existing cardiovascular disease, 7 reported a statistically significant association. The RR point estimates for mortality from COVID-19 and cardiovascular disease ranged from 0.72 to 8.89 (Fig 2). The overall pooled RR of COVID-19 mortality associated with cardiovascular disease was 2.25 (95% CI: 1.60–3.17), implying an approximately 2-fold greater risk of death. Between-study variation was moderate (I^2^ = 49, p = 0.02).

### The risk of mortality in patients with COVID-19 with 10 other pre-existing comorbidities

**Fig 3:**
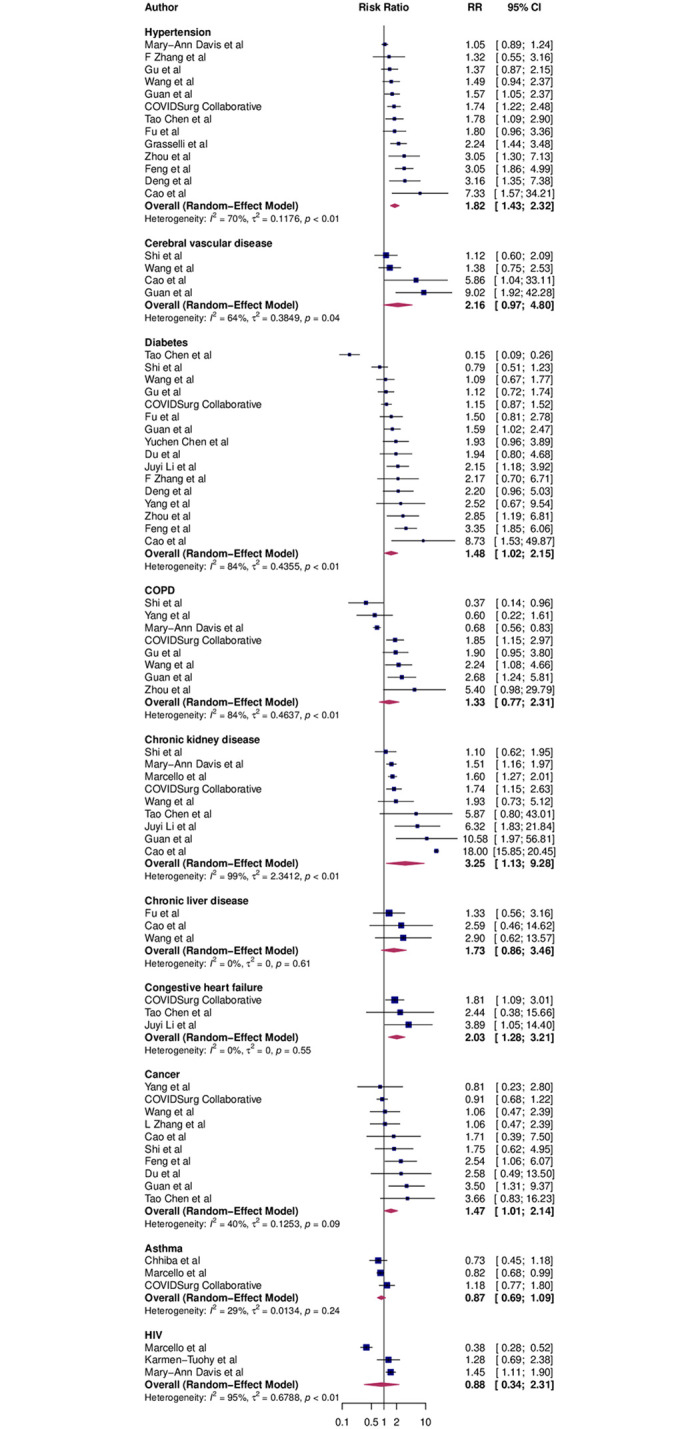
Association of 10 other comorbidities disease and mortality risk from COVID-19. Blue squares and their corresponding lines are the point estimates and 95% confidence intervals per each study. Maroon diamond represents the pooled effect estimate.

Compared with individuals without comorbidities, the risk of death was significantly greater in patients with hypertension (RR 1.82 [95% CI 1.43 to 2.32]), diabetes (1.48 [1.02 to 2.15]), congestive heart failure (2.03 [1.28 to 3.21]), chronic kidney disease (3.25 [1.13 to 9.28)]) and cancer (1.47 [1.01 to 2.14) (Fig 3). Cerebrovascular disease demonstrated a higher risk of death, though it was not a statistically significant finding (2.16 [95% CI 0.97 to 4.80]). In addition to this, chronic liver disease, COPD, asthma, and HIV/AIDS comorbidities were not significantly associated with greater risk of mortality.

### Publication bias, study heterogeneity, and meta-regression

The risk of publication bias was found to be significant for hypertension, cerebrovascular disease, and cancer (Table 2; the funnel plots are provided in S1 Fig). Trim and fill methods were used to adjust for the publication bias [22]. If the asymmetry is due to publication bias, our analyses suggest that the adjusted effect estimates for hypertension remained significant, However, the adjusted effect estimates for cancer were no longer significant.

**Table 2:** Estimated pooled RR of mortality from COVID-19 in patients with comorbidities compared to those without.

Comorbidity	N studies	Pooled effect size (95% CI), p-value	I^2^ (%), p-values	Egger’s test for publication bias (p-value)	Pooled effect size (95% CI), p-values after trim and fill*
**Cardiovascular disease**	14	2.25 (1.60 to 3.17), <0.0001	49%, 0.02	0.15	-
**Hypertension**	13	1.82 (1.43 to 2.32), <0.0001	70%, <0.0001	0.0006	1.29 (1.01 to 1.65), 0.04
**Diabetes**	16	1.48 (1.02 to 2.15), 0.04	84%, <0.01	0.15	-
**Congestive heart failure**	3	2.03 (1.28 to 3.21), 0.003	0%, 0.55	0.42	-
**Cerebrovascular disease**	4	2.16 (0.97 to 4.80), 0.06	64%, 0.04	0.04	1.23 (0.52 to 3.00) 0.62
**Chronic kidney disease**	9	3.25 (1.13 to 9.28), 0.03	99%, <0.0001	0.21	-
**Chronic liver disease**	3	1.73 (0.86 to 3.46), 0.12	0%, 0.61	0.16	-
**Cancer**	10	1.47 (1.01 to 2.14), 0.04	40%, 0.09	0.02	0.98 (0.66 to 1.46) 0.92
**COPD**	8	1.33 (0.77 to 2.31), 0.31	84%, <0.0001	0.12	-
**Asthma**	3	0.87 (0.69 to 1.09), 0.23	29%, 0.24	0.76	-
**HIV/AIDS**	3	0.88 (0.34 to 2.31),0.80	95%, <0.0001	0.97	-

**Abbreviations:** COPD: Chronic obstructive pulmonary disease; HIV/AIDS: Human immunodeficiency virus infection and acquired immune deficiency syndrome.

*Where publication bias was significant, trim and fill analyses were performed using the Duval and Tweedie non-paramteric method. [22]

Significant between- study heterogeneity was observed for cardiovascular disease, hypertension, diabetes, cerebrovascular disease, chronic kidney diseases, COPD, and HIV/AIDS. If a specific comorbidity had more than 10 studies and exhibited significant medium or high between-study heterogeneity, we further conducted meta-regression to explore the sources of heterogeneity. [46] Mean or median study age and the proportion of males were regressors in the univariate meta-regression. Results from the meta-regression analyses showed no statistically significant association between either mean study age or the proportion of males with estimated RRs for mortality (Table 3).

**Table 3:** Results of meta-regression analyses.

Comorbidity	Covariate*	N studies	Coefficient (95% CI)	p-value
**Cardiovascular**	% male	14	-0.003 (-0.066 to 0.060)	0.927
**Cardiovascular**	age	14	-0.015 (-0.072 to 0.042)	0.605
**Hypertension**	% male	13	0.007 (-0.016 to 0.029)	0.559
**Hypertension**	age	13	-0.001 (-0.035 to 0.032)	0.943
**Diabetes**	% male	16	-0.011 (-0.069 to 0.047)	0.706
**Diabetes**	age	16	-0.014 (-0.091 to 0.062)	0.716

*Age was mean age of study population, otherwise median was used.

We further performed influence sensitivity analyses for outcomes with high between-study heterogeneity by excluding and replacing one study at a time (Leave-One-Out method) from the meta-analysis and calculated the RR for the remaining studies [20]. No substantial change from any of the pooled RR were observed when other studies were removed in turn, indicating that no individual study had a considerable influence on the pooled estimate. The plots for the analysis estimates are provided in S2 Fig.

## Discussion

### Principal findings

The results of this systematic review and meta-analysis suggest that hospitalized patients with COVID-19 with pre-existing cardiovascular disease, hypertension, diabetes, congestive heart failure, chronic kidney disease and cancer have a greater risk of death from COVID-19.

### Strengths and limitations

This review provides up-to-date results of comorbidities’ influence on the higher risk of mortality in patients with COVID-19 by synthesizing a large number of recently published studies. The study yielded a large number of individuals from countries representing Europe, North America, Africa, and Asia. Nevertheless, this meta-analysis had several limitations. While some studies reported the race and ethnicity of study participants, others provided little or no information. Thus, we could not investigate race and ethnicity’s influence, which could have been a cause of variation between studies. Due to a lack of individual patient-level data, we used study level data in meta-regression analyses to assess heterogeneity between studies. We were unable to identify the main sources of heterogeneity in the effect estimates. Perhaps future analysis could use individual patient-level data to explore sources of heterogeneity.

Most of the studies included were at low risk of bias. Nevertheless, the NOS used to assess the included studies’ quality has low or unknown validity. [49] The alternative validated scale with high quality, the Risk of Bias in Nonrandomized Studies of Interventions (ROBINS-I), [50] could not be used in our meta-analysis because included studies did not involve any interventions. Nevertheless, we carefully evaluated included studies and listed potential confounders adjusted for each study that reported adjusted estimates. Furthermore, we investigated and reported adjusted effect estimates for the outcomes that showed potential publication bias (as quantified by a significant Egger’s test and asymmetrical funnel plots).

Many of the included studies reported effect size estimates from all covariates within each model, which can induce multiplicity and lead to “the Table 2 fallacy”. [51] From a causal inference perspective, this is a significant source of bias and could affect the interpretation of the results in causal terms. Many studies reporting COVID-19 mortality have been retrospective cohort studies or case-control studies that report odds ratios from logistic regression models, and yet COVID-19 mortality is relatively common (>10%). Since odds ratios of fairly common outcomes have the potential for biasing effect estimates towards a stronger association, the magnitude of the pooled estimates from our meta-analysis could be inflated. [52]

### Comparison with other studies

Our study findings are similar to results from previously published systematic reviews, suggesting a higher mortality rate of COVID-19 individuals with cardiovascular, chronic kidney disease and cancer. The overall 95% confidence interval obtained by Singh and colleagues overlapped with the one we identified. Pranata and colleagues assessed studies published until April 10^th^, 2020, and included 4448 patients from 16 studies. [53] They found a 2-fold greater risk in mortality in patients with COVID-19 with cardiovascular and cerebrovascular disease. In the current meta-analysis, the risk of mortality was approximately 2-fold higher in individuals with cerebrovascular disease. However, the association was not statistically significant. In addition to what is reported in published studies, this systematic review and meta-analysis incorporated evidence from the most recent studies, a large sample size, and a significant and higher estimated mortality risk of hypertension, diabetes, congestive heart failure, and chronic kidney disease than found previously.

A probable hypothesis for the pathophysiological mechanism related to the higher risk of mortality among patients with COVID-19 with pre-existing chronic conditions may stem from the increased allostatic load. Chronic conditions cause dysregulation of major physiological system, including the hypothalamic-pituitary-adrenal axis, the sympathetic nervous system, and the immune system. [54] The chronic nature of such conditions induces the ‘‘wear and tear” on the body’s regulatory system, [55] leading to the accumulation of pro-inflammatory cytokines, which affects the cellular immune system. As a result of the reduced immunity, these individuals become very susceptible to severe complications of SARS-CoV-2 and death. Such an association with this type of virus is not relatively new. Seasonal influenza, SARS-CoV, and Middle Eastern Respiratory Syndrome-CoV are also associated with increased severity and mortality in patients with preexisting conditions. [29, 56]

### Implications for research and clinical practice

As the race towards developing a vaccine against SARS-CoV-2 intensifies, our results support the notion that individuals who appear to be at increased risk should receive immunization priority. Individuals with pre-existing cardiovascular disease, hypertension, diabetes, congestive heart failure, chronic kidney disease and cancer are at heightened risk of death from the virus, thus should be given a priority during the allocation of a vaccine, especially if it is in limited supply. Historically, targeted public health vaccination intervention strategy for influenza vaccination is recommended by the Advisory Committee on Immunization Practices against seasonal influenza. [57] In the population with chronic comorbidities, annual influenza vaccination significantly reduces mortality and morbidity. [58] Mounting evidence postulates that SARS-CoV-2 may become seasonal requiring annual vaccination. [59]

The majority of patients with pre-existing cardiovascular disease, hypertension, diabetes, chronic kidney disease and congested heart failure use renin-angiotensin-aldosterone system (RAAS) blockers, which are postulated to increase the risk of developing a severe and fatal SARS-CoV-2 infection. [60] In experimental studies by Ferrario and colleagues, ACE inhibitors or ARBs increased cardiac levels of *Ace2* mRNA compared with a placebo. [61] Notably, cardiac levels of *Ace2* mRNA increased by 4.7-fold or 2.8-fold with either lisinopril (an ACE inhibitor) or losartan (an ARB), respectively. Nonetheless, recent clinical studies have found no association between the RAAS blockers and increased mortality from COVID-19. [62, 63] Such clinical or experimental evidence suggests that RAAS is an appropriate drug to manage hypertension and other cardiovascular diseases in the setting of COVID-19.

## Conclusion

Our findings suggest that of the major comorbidities analyzed, cardiovascular disease, hypertension, diabetes, congestive heart failure, chronic kidney disease, and cancer carry the highest risk of death from COVID-19. This research highlights the importance of paying attention to the populations with these specific comorbidities and tailor treatment to meet their need.

## Supporting information

S1 Table Risk ratios for the 11 comorbidities per study. (DOCX)

S1 Fig Funnel plots showing asymmetry. (DOCX)

S2 Fig Influential analysis. (DOCX)

S1 Checklist PRISMA checklist. (DOC)
