# Supplementary material for: Association of cardiovascular disease and 10 other pre-existing comorbidities with COVID-19 mortality: A systematic review and meta-analysis
Source: PLoS One. 2020 Aug 26;15(8):e0238215. doi: 10.1371/journal.pone.0238215 (PMC7449476; doi:10.1371/journal.pone.0238215)
Supplement: S1 Table — (DOCX) [file pone.0238215.s001.docx]

**Table S1: Risk ratios for the 11 comorbidities per study**

| Author | Congestive heart failure  RR (95%CI) | Cancer  RR (95%CI) | COPD  RR (95%CI) | Cardiovascular disease  RR (95%CI) | Diabetes  RR (95%CI) | Hypertension  RR (95%CI) | Chronic kidney disease RR (95%CI) | Chronic liver disease  RR (95%CI) | Cerebrovascular  disease  RR (95%CI) | HIV/AIDS  RR (95%CI) | Asthma  RR (95%CI) |
| --- | --- | --- | --- | --- | --- | --- | --- | --- | --- | --- | --- |
| Du et al |  | 2.58 (0.26 -26.04) |  | 2.464 (0.755 -8.044) | 1.94 (0.69 -5.45) |  |  |  |  |  |  |
| Zhou et al |  |  | 5.4 (0.96 -30.4) | 2.14 (0.26 -17.79) | 2.85 (1.35 -6.01) | 3.05 (1.57 -5.92) |  |  |  |  |  |
| Shi et al |  | 1.75 (0.43 -7.16) | 0.37 (0.04 -3.5) | 1.51 (0.7 -3.3) | 0.79 (0.41 -1.52) |  | 1.1 (0.49 -2.44) |  | 1.12 (0.46 -2.7) |  |  |
| Xiaochen Li et al |  |  |  |  |  |  |  |  |  |  |  |
| Guo et al |  |  |  | 5.99 (2.86 -12.55) |  |  |  |  |  |  |  |
| Grasselli et al |  |  |  |  |  | 2.24 (1.71 -2.95) |  |  |  |  |  |
| Tao Chen et al | 2.44 (0.15 -39.31) | 3.66 (0.7 -19.32) |  | 2.94 (1.58 -5.48) | 0.15 (0.07 -0.34) | 1.78 (1.14 -2.78) | 5.87 (0.65 -53.25) |  |  |  |  |
| Fu et al |  |  |  | 0.719 (0.189 -2.729) | 1.495 (0.717 -3.114) | 1.797 (0.941 -3.43) |  | 1.326 (0.375 -4.688) |  |  |  |
| Gu et al |  |  | 1.9 (0.9 -3.9) | 3 (1.8 -5) | 1.12 (0.64 -1.97) | 1.37 (0.83 -2.28) |  |  |  |  |  |
| Yang et al |  | 0.81 (0.07 -9.27) | 0.6 (0.08 -4.64) | 0.93 (0.14 -6.12) | 2.52 (0.47 -13.58) |  |  |  |  |  |  |
| Deng et al |  |  |  | 1.61 (0.66 -3.93) | 2.2 (0.93 -5.16) | 3.16 (1.67 -5.96) |  |  |  |  |  |
| Yuan et al |  |  |  |  |  |  |  |  |  |  |  |
| Wang et al |  | 1.06 (0.29 -3.86) | 2.24 (1.12 -4.45) | 1.86 (1.06 -3.26) | 1.088 (0.568 -2.084) | 1.494 (0.915 -2.438) | 1.93 (0.58 -6.48) | 2.902 (0.402 -20.943) | 1.38 (0.65 -2.93) |  |  |
| Juyi Li et al | 3.89 (1.1 -13.8) |  |  | 2.23 (1.12 -10.81) | 2.15 (1.29 -3.58) |  |  |  |  |  |  |
| L Zhang et al |  | 1.06 (0.29 -3.86) |  |  |  |  |  |  |  |  |  |
| Feng et al |  | 2.54 (1.11 -5.81) |  | 4.96 (3.96 -6.21) | 3.35 (2.65 -4.25) | 3.05 (2.56 -3.62) |  |  |  |  |  |
| F Zhang et al |  |  |  |  | 2.17 (0.53 -8.93) | 1.32 (0.37 -4.73) |  |  |  |  |  |
| Guan et al |  | 3.501 (1.604 -7.643) | 2.681 (1.424 -5.048) | 5.56 (2.48 -12.45) | 1.586 (1.028 -2.449) | 1.575 (1.069 -2.322) | 10.58 (3.71 -30.15) |  | 9.02 (3.51 -23.23) |  |  |
| Yuchen Chen et al |  |  |  |  | 1.93 (0.93 -4) |  |  |  |  |  |  |
| Cao et al |  | 1.71 (0.17 -17.48) |  | 8.89 (1.36 -58.09) | 8.73 (2.28 -33.46) | 7.33 (2.37 -22.65) | 18 (1.75 -1.85) | 2.59 (0.22 -30.34) | 5.86 (1.07 -31.99) |  |  |
| COVIDSurg Collaborative | 1.81 (1.13 -2.89) | 0.91 (0.63 -1.33) | 1.85 (1.23 -2.79) |  | 1.15 (0.85 -1.58) | 1.74 (1.31 -2.3) | 1.74 (1.22 -2.49) |  |  |  | 1.18 (0.70-1.98) |
| Chhiba et al |  |  |  |  |  |  |  |  |  |  | 0.73 (0.35-1.55) |
| Marcello et al |  |  |  |  |  |  |  |  |  | 0.38 (0.26 -0.55) | 0.82 (0.66 -1.04) |
| Karmen Tuohy et al |  |  |  |  |  |  |  |  |  | 1.28 (0.57 -2.9) |  |
| Mary-Ann Davis et al |  |  | 0.68 (0.53 -0.86) |  |  | 1.05 (0.88 -1.27) | 1.51 (1.2 -1.89) |  |  | 1.45 (1.14 -1.84) |  |
