# Supplementary material for: Association of cardiovascular disease and 10 other pre-existing comorbidities with COVID-19 mortality: A systematic review and meta-analysis
Source: PLoS One. 2020 Aug 26;15(8):e0238215. doi: 10.1371/journal.pone.0238215 (PMC7449476; doi:10.1371/journal.pone.0238215)
Supplement: S1 Fig — (DOCX) [file pone.0238215.s002.docx]

**Figure S1; Funnel plots showing asymmetry**

Cancer

Cerebrovascular disease

Hypertension
